# Understanding security challenges in the software supply chain through causal relationships

**DOI:** 10.1371/journal.pone.0344098

**Published:** 2026-03-05

**Authors:** Aylin Adem, Erman Çakıt, Metin Dağdeviren, Beata Mrugalska, Waldemar Karwowski

**Affiliations:** 1 Department of Industrial Engineering, Gazi University, Ankara, Türkiye; 2 Faculty of Engineering Management, Institute of Safety and Quality Engineering, Poznan University of Technology, Poznań, Poland; 3 Department of Industrial Engineering and Management Systems, University of Central Florida, Orlando, Florida, United States of America; Istanbul University: Istanbul Universitesi, TÜRKIYE

## Abstract

In recent years, the Software Supply Chain (SSC) has become a key target for cyberattacks due to its complex structure and dependence on third-party and open-source components. These attacks pose serious risks to the integrity and security of software systems. While many studies have explored solutions to specific security issues in the SSC, the relationships among the barriers to achieving robust security have not been comprehensively analyzed—particularly in the context of SSC security challenges using the Decision-Making Trial and Evaluation Laboratory (DEMATEL) technique. This study addresses this gap by identifying and analyzing the major challenges that weaken SSC security. To do this, the DEMATEL method was used to explore how different security challenges affect each other. Ten key challenges were identified based on a detailed literature review. The findings indicated that the three most significant challenges are insecure software distribution mechanisms, inadequate continuous monitoring and incident response capabilities, and the growing complexity and diversity of cyber-attacks. By visualizing the relationships between these challenges, this study clarifies where to focus security efforts. Solving root causes can lead to broader improvements across the entire software supply chain. The findings offer practical insights for decision-makers seeking to improve cybersecurity strategies in software development environments.

## Introduction

Software complexity arises not just from the code written within a specific project, but also from the extensive network of direct and transitive dependencies it relies on. A supply chain is a global system that delivers goods and services to consumers through coordinated flows of data, distribution, and money [1]. The supply chain serves as the foundation of today’s consumer-driven world. Every product on the market passes through a series of interconnected stakeholders who coordinate in complex ways to deliver the end product. Modern supply chains extend across diverse geographic regions and socioeconomic contexts, each necessitating tailored checks and controls to maintain seamless operations.

Moreover, advancements like the Internet of Things (IoT) and 5G have greatly enhanced the efficiency and effectiveness of supply chain management [2]. In today’s increasingly digital and interconnected world, the Software Supply Chain (SSC) has emerged as a critical focal point for both innovation and security [3]. Traditionally associated with manufacturing and logistics, the concept of a supply chain has been adapted to the software domain to describe the ecosystem of tools, processes, dependencies, and contributors involved in the development, delivery, and maintenance of software products. In recent years, there has been a notable surge in attacks targeting the SSC, prompting increased attention and concern from both industry and government sectors [4]. The SSC has become a vital and increasingly intricate element of modern software development [5]. Unlike traditional models where most code was developed internally; today’s software is largely built from numerous external sources—chiefly open-source libraries and third-party tools [6]. While this component-based approach accelerates innovation, it also brings heightened risks, including those typical of conventional supply chains, such as delays, counterfeit elements, human errors, and various internal and external threats [7]. Moreover, it introduces complex challenges related to integrity, governance, and security—particularly the risk of vulnerabilities in the code that could be exploited [8]. However, despite widespread recognition of these issues, there is a notable lack of structured frameworks that explore how these numerous challenges interact, reinforce, or mitigate each other. Existing discussions often treat these barriers as isolated problems without systematically analyzing their interdependencies, leaving organizations without clear guidance on which issues to prioritize to maximize security impact. In other words, software developers did not foresee the SSC becoming a deliberate target for attacks. The industry has shifted from dealing with passive threats that exploit unintended vulnerabilities left by well-meaning developers to facing a new wave of supply chain attacks that actively target developers themselves, circumventing traditional security measures. These attackers now embed malicious code directly into open-source components and compromise build and deployment pipelines. Securing the SSC requires not only the implementation of robust technical safeguards but also a comprehensive understanding of the systemic and often deeply embedded barriers that hinder effective protection throughout the chain [9]. These barriers are multifaceted and interwoven, spanning both technical and socio-organizational dimensions. Common challenges include the lack of standardized regulations and industry-wide security protocols, limited financial and human resources, weak configuration management practices, insecure software distribution mechanisms, and critical vulnerabilities introduced by human error or malicious intent. Additionally, inadequate continuous monitoring and incident response capabilities, insufficient authorization and access control measures, unregulated use of open-source components and absence of a comprehensive Software Bill of Materials (SBOM), outdated or unmanaged software dependencies, and the growing complexity and diversity of cyber-attacks further exacerbate the threat landscape. This complex environment creates an urgent need to identify not just the list of barriers but also the causal relationships among them. Without such insights, organizations may allocate resources to symptoms rather than root causes, undermining their security efforts. These interconnected factors create a dynamic and often opaque risk environment, where isolating the root causes of vulnerabilities or determining the most effective mitigation strategies becomes a significant challenge. As a result, organizations may struggle to allocate resources appropriately or develop coherent, proactive security strategies. In this context, Multi-Criteria Decision-Making (MCDM) approaches offer a valuable framework for disentangling and systematically evaluating the intricate relationships among these barriers. MCDM methods offer a structured framework that integrates decision-makers’ viewpoints to address a wide range of complex problems [10]. Today, MCDM approaches are increasingly used to tackle both practical and theoretical challenges, either individually or through hybrid models. Specifically, the Decision-Making Trial and Evaluation Laboratory (DEMATEL) method enables the identification and analysis of causal and effect relationships among variables, helping to distinguish between factors that drive risk and those that are primarily impacted by it [11]. Previous studies on cybersecurity and supply-chain risk frequently relied on MCDM methods like AHP, ISM, ANP, and TOPSIS. While these tools are effective for prioritizing risks or structuring factor relationships, they fall short when analyzing the highly dynamic and interdependent nature of SSC security challenges. Methods like AHP and TOPSIS assume simple, linear or hierarchical relationships, preventing them from capturing the bidirectional causality common in SSCs [12,13]. ISM can map structural dependencies but fails to quantify the strength of influence [14]. ANP, while handling interdependencies, requires predefined alternatives and is not ideal for exploratory causal discovery [15].

Given these shortcomings, existing MCDM techniques are insufficient for analyzing the complex, non-linear, and mutually reinforcing interactions found in SSC vulnerabilities. In contrast, the DEMATEL method is uniquely suited for this domain. It is specifically engineered to model cause-and-effect relationships in complex systems by quantifying both direct and indirect influences. DEMATEL’s ability to distinguish root causes from effects, create detailed causal impact maps, and capture the systemic behavior of interdependent challenges makes it exceptionally appropriate for SSC analysis. By applying DEMATEL to the SSC context, this study fills a crucial gap in the literature by systematically revealing the underlying causal interrelationships among key security barriers.

By applying the DEMATEL method to the SSC context, this study fills a crucial gap in the literature by systematically uncovering the causal interrelationships among key security barriers. This analysis highlights the most influential factors, enabling stakeholders to prioritize efforts, allocate resources more effectively, and implement targeted interventions. Ultimately, the findings aim to support evidence-based decisions that enhance risk management and bolster the overall resilience of software supply chains.

The presented study was aimed at addressing the following research questions:

- **RQ1:** What are the key security challenges affecting software supply chain security as identified in the literature and expert evaluations?
- **RQ2:** How do these challenges influence one another, and which of them function as root causes versus resulting effects within the SSC ecosystem?
- **RQ3:** Which challenges exert the strongest causal influence on SSC security, and therefore should be prioritized in mitigation strategies?

The rest of this study is organized as follows: Section 2 presents a review of the relevant literature, while Section 3 offers a detailed explanation of the DEMATEL method. Section 4 presents the implementation of the DEMATEL method along with a comparative analysis of the results. Section 5 discusses the practical aspects of the study’s implementation. Finally, the conclusion summarizes the key findings, study limitations, and offers suggestions for future research.

## Literature review

In the literature, the topic of SSC has been addressed in various ways. In addition to conceptual framework studies that aim to bring academic maturity to the field, there are also practical studies focused on developing applications to directly enhance security. Furthermore, the literature includes survey-based or short interview studies aimed at understanding experts’ perspectives on SSC security. Building on this perspective, the literature review has been structured to reflect these diverse approaches.

### Security enhancement approaches in SSC

This section examines recent studies focused on strengthening SSC security, with particular attention to risks arising from third-party components, dependencies, and development processes. Jia et al [16] examined how function inlining affects four key security tasks within the SSC: code search, open-source usage detection, vulnerability detection, and patch presence testing. Their findings showed that many existing studies neglect function inlining and rely on a one-to-one matching approach. This omission results in a performance drop of 30% in code search and 40% in vulnerability detection—both critical security tasks in the SSC. Additionally, Jia et al [16] noted that most inlined functions were ignored in the other two tasks, leaving these functions exposed to potential risks. Based on their findings, they proposed conditional inlining and incremental inlining as part of a low-cost, high-coverage inlining strategy.

Zhou et al. [17] proposed an improved platform based on blockchain technologies and microservice architecture for Software Composition Analysis (SCA), aiming to enhance SSC security using SBOM. The system proposed by the authors seeks to improve SSC security by providing an SBOM sharing platform with reliable data integrity, guaranteed tool security, and good interoperability. Wang et al. [18] proposed a new detection model for identifying Algorithmic Complexity Vulnerability (ACV), which is likely to cause security problems in the SSC, especially due to the use of third-party components. They stated that existing tools in the literature detect ACV by relying on abstract loop and iterative patterns and by fuzzing the entire application. The authors reported that by testing the model they developed on open-source third-party components, they achieved faster performance, higher efficiency, and greater accuracy compared to existing tools when detecting security vulnerabilities. Xu et al. [19] developed GRINGOTTS, an end-to-end encrypted Version Control System (VCS), to eliminate the existing security disadvantages of current VCS, which play an important role in the SSC and to make them more secure. To verify whether their system improved security, they implemented it in different code projects and demonstrated increased security through various experiments. Wang et al [20] worked on the static detection of security vulnerabilities in binary programs. They proposed BinVulDet to detect security vulnerabilities, an important research area in SSC security. BinVulDet detects security vulnerabilities through dependencies by using decompilation techniques, program slicing techniques, and a neural network. The authors stated that their developed tool achieved better results than existing binary vulnerability detection methods. Marjanović et al. [21] pointed out that the use of third-party code is an important risk parameter when evaluating whether all suppliers have a secure software development lifecycle process in SSC security. In this study, they worked on a decentralized, immutable, blockchain-based approach that provides reliable visibility of security development lifecycle metrics over a certain period. Wang, Wu and Lou [22] addressed proposing a comprehensive portrait model to prevent security-related problems in the SSC. This model consists of a threat model and a threat surface indicator system, and it contains multiple levels and dimensions. The portrait model is based on a generative artificial intelligence model. They tested the portrait model they developed on major SSC security problems (e.g., SolarWinds, PHP Backdoor) and presented the effectiveness of the model. Imtiaz and Williams [23] aimed to help developers safely accept dependency updates by measuring whether code changes go through the code review process in an update. They noted that security issues are encountered in the SSC due to the use of open-source code and updates. Based on this, they conducted an experimental study using Depdive, an update control tool, and highlighted that unreviewed code can be added to the SSC during updates, potentially causing security issues.

Soto Volera et al. [24] stated that using third-party code in the SSC creates serious difficulties in terms of maintenance and security. They focused on the dependencies in this context and proposed a technique, called DEPTRIM, that automatically analyzes the dependencies, permits only those necessary for the software project, and removes the remaining ones. They claimed that this approach resulted in significantly less third-party code being used compared to the software project’s initial version. They demonstrated the effectiveness of their technique by testing it with open-source Java projects. In their studies, Nahum et al. [25] stated that open-source software (OSS) is preferred in the supply chain due to its economic value, but OSS can pose a security threat to the supply chain as an attack tool. They mentioned that OSS endangers the security of the system by injecting malicious code into target libraries. They proposed a security framework called Secure Crowd-Sourced Code Verification to prevent targeted OSS attacks against specific developers in the supply chain. They integrated the framework into the early stages of software production to serve as a code verification step. They reported that, in tests involving nearly 900 OSS projects, their framework provided very prompt warnings in the event of any security breach. Hammi and Zeadally [1] discussed the similarities and differences between the supply chain structures of physical products and software. They argued that each link connecting the elements in the supply chain is vital for the overall system’s security. They developed a supply chain model that provides protection for each link in the SSC and conducted formal validation to verify the robustness and security of the model. Axelrod [26] aimed to facilitate the categorization of SSC by extending the Cynefin framework, a decision-making model used for solving complex problems. Based on this, the author commented on how precautions should be taken against risks in each category. He emphasized that, since all newly developed systems will rely on SSC, the obstacles to ensuring the security of these systems must be overcome. Scacchi and Alspaugh [27] stated that each software has architectural models, and that these models can be visually mapped, which helps ensure the security of SSC. They presented a visual model of an open architecture ecosystem and explained the threats in SSC, as well as the defense mechanisms developed against them based on the SSC process and open architecture (OA). They noted that OA ecosystem maps can serve as models of the software system, thereby revealing the relationships between software components. Additionally, they pointed out that since data flow and control paths are visible in OA maps, threats can be easily detected. The authors argued that OA ecosystem maps can play an important role in identifying and preventing security risks in SSC. Liang et al. [28] stated that the development of smart transportation systems has made the use of open-source code inevitable. Since it is known that using open-source code creates security problems in SSC there has been a focus on studies, especially in the transportation industry. The authors proposed an open-source governance platform specific to the transportation industry and presented its technical map. They argued that security risks can be mitigated by using this platform. Bi et al. [29] emphasized that SBOMs are an important security solution in SSC. They investigated the problems and solutions of using SBOMs to enhance security through repository mining and descriptive analysis in 510 SBOM-related projects. They analyzed the life cycle of SBOM by dividing it into main and sub-stages as well. Tsfaty and Fire [30] developed an algorithm for detecting malicious code integrated into open-source code in SSC. They tested the detection algorithm, which is based on deep learning, on various datasets and stated that its performance was satisfactory and applicable to real-world problems. Ladisa et al. [31] proposed a taxonomy to classify attacks on SSC and to create a common terminology. They also proposed a visualization tool called Risk Explorer. They stated that this tool can help with threat modeling, scope red team activities, and gap analysis of safeguards in open-source SSC. In their study, Doumanidis et al. [32] stated that Industrial Control Systems (ICS) are systems that perform critical control processes in the regulation of industrial operations. They also mentioned that the use of third-party open-source code in these systems makes the SSC vulnerable to threats. They selected examples from ICS vendors, chose device alternatives and PLC firmware, and analyzed them using tools such as Binwalk, Cutter, and Changelog. Through this, they aimed to understand how vendors select, install, or remove third-party components in their software. Although the use of third-party code is widespread in ICS, they concluded that delayed updates and security issues in third-party components could threaten the entire system. They also suggested that ICS vendors should publish SBOMs to mitigate these risks.

### Survey and interview-based research

This section summarizes survey and interview-based studies that investigate security challenges in the SSC from organizational and practitioner perspectives. Enck and Williams [33] the security challenges of SSC by conducting interviews and observations involving 30 large corporations from both industry and the U.S. government. Determined challenges of SSC security are updating vulnerable dependencies, leveraging the SBoM for security, choosing trusted supply chain dependencies, securing the build process, getting industry-wide participation. Voas and Hurlburt [34] studied explaining and analyzing some security problems in software development using the elements of SSC. They stated that the presence of third parties in software development brings additional responsibility for evaluators regarding security problems and developed a software and system assurance model. They addressed the necessary function definitions related to this model.

### Empirical and conceptual guidance

This section presents empirical and conceptual studies that provide guidance on understanding and mitigating security risks in the SSC. Arora et al. [35] introduced the concept of SBoM from all aspects. They conducted two case studies in their research, focusing on its use for security improvements in the SSC, specifically the SolarWinds attack and the Log4j vulnerability. They provided recommendations to researchers and tool developers regarding SBoM and offered a roadmap for building an effective SBoM. Jackson [36] stated that there are security risks due to the use of open-source code in SSC. He suggested a guide consisting of four stages and stated that the risks arising from the use of open-source code in SSC would be reduced. Axelrod [37] classified the risks in SSC according to the origin of the software (i.e., open source or custom). The author particularly stated that using simulations is essential to fully understand the complex structure of SSC and analyze security risks. Martinez and Duran [38] analyzed the SolarWinds case in their study based solely on articles written about SolarWinds in the literature. They pointed out the risks to supply chain security brought using open-source code and suggested the use of tools such as Zero Trust and Multi-Factor Authentication (MFA) mechanisms to ensure security in the supply chain. Melara and Torres-Arias [39] stated that with the widespread use of the Internet in software development it has become common for software developers from all over the world to develop new software using open-source code from other developers. Based on this, they emphasized the need to establish a non-local common language to prevent security risks in the SSC. Additionally, they noted that similarities and differences between physical and digital supply chains should be systematically analyzed, and new security techniques should be developed rather than relying on existing ones (such as SBOM) to ensure the security of the SSC. It may not be possible to detect an attack on the SSC until it performs one of its tasks, such as gathering information or sabotaging the system and rendering it inoperable. This is especially concerning for healthcare devices (devices used at home that transfer data by connecting to the cloud), as security risks in the SSC can also harm the end user—the human. Wirth [40] addressed these issues in his study and stated that attacks on the SSC are conducted through the app store by embedding malicious code in open-source software or by infiltrating secure certificates. In his study, Wirth also proposed several precautions against SSC attacks in healthcare systems. Takayuki et al. [41] emphasized that SBOM plays an important role in ensuring security in SSC. However, special attention should be paid to software dependencies and the management and use of big data to further enhance its effectiveness. Researchers stated that they are working on supply chain security risk management based on the key concept of security transparency technology.

As a result of the comprehensive literature review (see Table 1), it is evident that the SSC management concept has reached a sufficient level of maturity. Numerous studies have been conducted on this subject, and in these studies, the existence of security-related problems and various approaches to address them have been suggested. However, a holistic perspective has not been adequately emphasized. In particular, although the obstacles to creating the necessary conditions for ensuring the security of SSCM have occasionally been mentioned, a comprehensive analysis of these obstacles has not been conducted.

**Table 1:** Summary of Literature.

Category	Authors	Approach	Key Contribution
**Security Enhancement Approaches in SSC**	Jia et al. [16]	Conditional & Incremental Inlining	Resolved inlining-related performance issues (code search and vulnerability detection) with a low-cost, high-impact solution
	Zhou et al. [17]	Software Composition Analysis	Improved data integrity, tool security, and interoperability in blockchain-based SBOM platform
	Wang et al. [18]	Algorithmic Complexity Vulnerability Detection	Achieved faster, more efficient, and accurate vulnerability detection
	Xu et al. [19]	GRINGOTTS (Encrypted Version control security)	Improved VCS security through encryption and practical experiments
	Wang et al. [20]	BinVulDet (Static binary vulnerability detection)	Employed decompilation, slicing, and neural networks to outperform existing methods.
	Marjanović et al. [21]	decentralized, immutable, blockchain-based approach	Providing reliable visibility of security development lifecycle metrics over a certain period
	Wang, Wu, Lou [22]	AI-based threat modelling	Validated model with real-world attacks (e.g., SolarWinds)
	Imtiaz & Williams [23]	Depdive Update control tool	Highlighted risks of unreviewed dependency updates in SSC and the importance of dependency updates & code review
	Soto Volera et al. [24]	DEPTRIM (Dependency trimming)	Reduced third-party code minimization and unnecessary dependencies.
	Nahum et al. [25]	Secure Crowd-Sourced Code Verification	Framework for early-stage malicious code detection
	Hammi & Zeadally [1]	Secure SSC model developing	Developed a validated model protecting each SSC link
	Axelrod [26]	Extended Cynefin framework	Categorized risks and suggested strategies for SSCs
	Scacchi & Alspaugh [27]	Open Architecture ecosystem maps	Visualized component relationships and control paths to improve SSC security
	Liang et al. [28]	Domain-specific Governance platform	Mitigated OSS risks in smart transportation systems
	Bi et al. [29]	SBOM lifecycle analysis (repository mining and descriptive analysis)	Analyzed over 500 projects to understand structured SBOM lifecycle and security benefits
	Tsfaty & Fire [30]	Deep learning–based malicious code detector	Demonstrated real-world applicability of developed detection algorithm
	Ladisa et al. [31]	SSC attack taxonomy & Risk Explorer	Provided common terminology and tools for threat visualization
	Doumanidis et al. [32]	Software analysis using Binwalk, Cutter, and Changelog	Identified vulnerabilities due to third-party code in that Industrial Control Systems
**Survey and Interview-Based Research**	Enck & Williams [33]	Industry interviews	Identified five key SSC security challenges from 30 entities.
	Voas & Hurlburt [34]	Security responsibility in SSC	Modeled necessary evaluator functions due to third-party involvement
**Empirical and Conceptual Guidance**	Cox [6]	Open-Source Software Supply Chain Security	Proposed precautions can be taken against the risk of using open-source SSC
	Arora et al. [35]	SBOM-focused case studies (SolarWinds, Log4j)	Offered roadmap and recommendations for SBOM usage.
	Jackson [36]	Four-stage risk reduction guide	Provided practical guidance for minimizing OSS-related risks
	Axelrod [37]	Risk categorization	Emphasized simulations to understand SSC complexity and risks
	Martinez & Duran [38]	Case-based SSC risk analysis	Advocated for Multi Factor Authentication and Zero Trust tools to prevent similar attacks.
	Melara & Torres-Arias [39]	SSC risks	Called the need for non-local SSC security language and new techniques beyond SBOM
	Wirth [40]	SSC attack paths	Warned against app store and certificate-based attacks; suggested precautions
	Takayuki et al. [41]	SBOM	Focused on dependency and big data management for improved SSC security.

To the best of our knowledge, previous studies have not examined the interrelationships among these obstacles by comprehensively addressing the security challenges of software supply chain management and applying the DEMATEL technique. By employing DEMATEL, our study provides a structured understanding of the effects and interactions among SSC security challenges. This approach enables stakeholders across the software supply chain to identify critical issues and make informed, strategic decisions when implementing preventive measures against security vulnerabilities.

The complexity and multifaceted nature of SSC security challenges make traditional analytical methods inadequate. A more suitable technique must be able to (i) identify the direction in which one challenge influences another and (ii) quantify the strength of these relationships. The DEMATEL method meets this requirement by effectively modeling cause-and-effect relationships within a system and graphically illustrating the direct and indirect pathways through which factors exert influence. The DEMATEL technique is distinguished from other multi-criteria decision-making methods by its detailed computational framework for capturing interrelationships among elements. By contrast, the AHP technique is based on the assumption that the elements are independent and yields only a ranking; hence, it was not adopted in this study. This unique capability allows researchers to clearly differentiate root causes from resulting effects, which is crucial for developing specific and effective prevention strategies within the SSC context.

This study aims to fill these gaps in the literature by providing a thorough analysis of the interactions and dependencies among the obstacles to SSCMS, as well as the contexts in which they influence each other. To this end, the DEMATEL technique was employed to analytically examine the relationships among SSCMS obstacles, and the dependencies were visualized using relational diagrams.

## DEMATEL (decision-making trial and evaluation laboratory)

Multi-Criteria Decision-Making (MCDM) techniques follow a systematic approach that incorporates the perspectives of decision-makers in addressing diverse and complex problems [42]. These techniques, which may be applied individually or in an integrated manner, are adapted according to the specific characteristics of the problem to be solved [43,44]. Today, MCDM methods are increasingly utilized in the resolution of both theoretical and practical issues, either separately or in combination. Among these methods, the Decision-Making Trial and Evaluation Laboratory (DEMATEL) is employed to analyze the interrelationships among criteria that influence the decision-making process, specifically to determine whether these criteria affect one another and whether they are independent or interdependent [45].

The DEMATEL method was developed by Fontela and Gabus [46] in 1974 to analyze complex and interrelated problems. Recognized in the literature as one of the most effective tools for identifying cause-and-effect relationships among evaluation criteria [47,48], this technique has been widely adopted and frequently applied across various fields [11,49,50]. DEMATEL is capable of converting the relationships among factors into a clear structural model of the system, categorizing the factors into cause-and-effect groups [51]. One of the advantages of DEMATEL is its ability to visually represent the relationships between criteria [52].

One of the primary objectives of this paper is to analyze the interrelationships among the identified SSC security challenges. The DEMATEL technique is particularly well-suited for this purpose, as it effectively distinguishes between cause-and-effect groups within a set of criteria. Unlike other MCDM methods such as AHP, which can only assign weights to the security challenges without revealing the relationships among them, DEMATEL offers valuable insights into the structure of these interdependencies. Similarly, ANP is not appropriate in this context, as the relationships among the security challenges have not yet been established. Moreover, ANP requires defined alternatives related to the decision-making problem in order to construct a supermatrix and solve the decision-making problem, which do not exist for this paper. As there is no uncertainty in the data structure or ambiguity in expert evaluations, the traditional DEMATEL method using crisp values was applied. The complete calculation process of the DEMATEL method is outlined as follows [45,46,52]:

The initial step in the DEMATEL method involves constructing the direct relationship matrix. In this stage, the relevant matrix is obtained through pairwise comparisons between criteria, where 𝑧𝑖𝑗 indicates the degree to which criterion ci influences criterion c_j_. During the construction of this matrix, the scale provided in Table 2 is used.

**Table 2:** The evaluation scales.

Linguistic expression	Numerical value
No influence	0
Low influence	1
Medium influence	2
High influence	3
Very high influence	4

The process begins by constructing the individual direct-relation matrix 𝑍_𝑘_ = [𝑧_𝑖𝑗_^k^] _𝑛×𝑛_ based on the assessment of the 𝑘th expert. By aggregating the opinions of the m experts, the group direct-influence matrix 𝑍 = [𝑧𝑖𝑗]×𝑛 can be obtained by applying the (1), which is a simple arithmetic aggregation:


$$\begin{array}{c} z_{ij}=\frac{\text{1}}{m}\sum {\mathrm{\backslash nolimits}}_{k=\text{1}}^{l}{z_{ij}}^{k}\;i,j=\text{1},\text{2},\ldots .,n. \end{array}$$ (1)

After gaining the aggregated direct relation matrix, now the normalized direct-relation matrix (D =[D_𝑖𝑗_] _×𝑛_) can be calculated. Eq. (2) and Eq. (3) are used for this calculation.


$$\text{D}=\frac{Z}{s}$$ (2)

$$\begin{array}{c} s=\text{max}(\text{max}\sum {\mathrm{\backslash nolimits}}_{j=\text{1}}^{n}z_{ij},\;\text{max}\sum {\mathrm{\backslash nolimits}}_{i=\text{1}}^{n}z_{ij})\; \end{array}$$ (3)

Afterward, total relation matrix T = [t_𝑖𝑗_]_×𝑛_ need to be calculated based on the values of D matrix. In order to achieve T matrix, one has to apply the following formula:

𝑇 = D + D^2^ + D^3^ + ⋅ ⋅⋅ + D^ℎ^ = D((𝐼 −  D)^−1^, as ℎ →∞, where 𝐼 represents the identity matrix. The next step of the application of DEMATEL technique is to generate the influential relation map (IRM). For gaining this map, the vectors 𝑅 ([r_𝑖_]_×1_) and 𝐶 ([c_j_]_1×n_) must be calculated, with the rows and columns of the total-relation matrix 𝑇 being summed, respectively, as defined by the (4) and (5):


$$\begin{array}{c} R=\sum {\mathrm{\backslash nolimits}}_{j=\text{1}}^{n}(tij) \end{array}$$ (4)

$$\begin{array}{c} C=\sum {\mathrm{\backslash nolimits}}_{i=\text{1}}^{n}(tij) \end{array}$$ (5)

In the stage of visually displaying the interactions between the criteria, which is one of the most important advantages of DEMATEL, it is necessary to determine to which group (affected or affecting) the relevant criteria belong. The following procedure is followed for this decision:

The sum of (𝑅 + 𝐶), known as “Importance,” represents the degree to which a factor plays a central role in the system. Similarly, the vertical axis vector (𝑅 − 𝐶), referred to as “Relationship,” shows the net effect a factor has on the system. If (𝑟𝑗 − 𝑐𝑗) is positive, it indicates that factor 𝑗 exerts a net effect on other factors, categorizing it in the cause group. On the other hand, if (𝑟𝑗 − 𝑐𝑗) is negative, factor 𝑗 is primarily influenced by other factors and is therefore placed in the effect group.

If desired, the weights of the criteria — that is, their relative priorities — can also be determined using the DEMATEL method. For this purpose, (6) is applied:


$$\text{wj}=\frac{\sqrt{{\left(\text{rj}+\text{ cj}\right)}^{\text{2}}+{\left(\text{rj }-\text{ cj}\right)}^{\text{2}}}}{\sum_{j=\text{1}}^{n}\sqrt{{\left(\text{rj}+\text{ cj}\right)}^{\text{2}}+{\left(\text{rj }-\text{ cj}\right)}^{\text{2}}}}$$ (6)

Finally, the threshold value is determined. Particularly in cases where the number of criteria is high, considering all relationships among them may result in an overly complex influence map, which can hinder clear interpretation and effective decision-making. If the threshold value is set too low, the resulting network may become excessively dense and difficult to interpret, whereas an overly high threshold may exclude relevant relationships and thus limit meaningful system analysis [53]. Therefore, applying a threshold value and visualizing only relationships that exceed this level is a widely accepted approach to simplify the analysis and highlight the most significant interactions. In this study, the threshold value was determined as the mean of the elements of the total-relation (T) matrix, in line with established practices in DEMATEL-based studies [54–57]. This mean-based approach is commonly adopted to achieve a balanced representation that preserves essential information while ensuring network clarity and interpretability. It should be noted that in DEMATEL analyses, the threshold value is not a statistically estimated parameter but rather a methodological filtering mechanism used to eliminate negligible causal relations and improve the interpretability of the influence network. Accordingly, threshold values are typically determined based on expert judgment or matrix-derived values, rather than formal statistical testing. Consistent with a large body of DEMATEL literature, the threshold value in this study was defined as the mean of the elements of the total-relation matrix. This approach has been widely adopted as a standard practice to balance information retention and network clarity, ensuring that only meaningful causal relationships are visualized while avoiding excessive network density. Regarding sensitivity analysis, DEMATEL is primarily an exploratory structural modeling technique aimed at identifying causal relationships rather than estimating precise numerical effects. Variations in the threshold value influence only the density and visualization of the causal diagram and do not affect the calculated influence degrees or the cause–effect classification of factors. Consequently, sensitivity analysis with respect to the threshold value is not commonly reported in DEMATEL applications and is not expected to provide additional insight into the underlying causal structure. Given these methodological considerations and established practices in the literature, the use of a mean-based threshold value is considered appropriate and sufficient for the objectives of this study.

## Application and results

The first step of the application is to identify the challenges. Based on the results of the literature review, some groupings were made to ensure that the number of identified criteria was large enough to be comprehensive, but not excessively so. The identification process is based on a systematic literature review (see Table 1), which shows that SSC security challenges mainly fall into regulatory, technical, organizational, and human-related dimensions. The ten challenges (C1–C10) were selected based on their prominence in the literature, their direct impact on SSC security, and their interrelationships. Specifically, C1 addresses regulatory and governance deficiencies; C2 organizational capacity and resource constraints; C3–C4 technical process weaknesses; C5–C7 human-related access and authorization risks; C8–C9 issues related to open-source use, SBOM deficiencies, and dependency management; and C10 increasing attack complexity and uncertainty. Together, these challenges provide a holistic view of SSC security. Since the aim is to provide an overall view of the process, supplier reliability or supply chain transparency were not treated as separate criteria; instead, the study focuses on the challenges that may arise and need to be addressed in order to ensure software supply chain security. The ten literature-supported criteria comprehensively cover the SSC process while minimizing conceptual overlap. Accordingly, no key security challenge was excluded; rather, all were systematically organized into a structure suitable for causal relationship analysis Table 3.

**Table 3:** The determined challenges and explanations.

Challenges	Explanations
(C1) Lack of standardized regulations and industry-wide security protocols	Since software security developments are still ongoing, there is currently no universally accepted regulation or guideline regarding the measures that need to be taken to ensure security on a national or global scale. This includes the processes to be followed when software developers release their code as open source, distribute it, and it is subsequently used by different institutions. This lack of regulation is identified as an obstacle to ensuring the security of the SSCS. The absence of a comprehensive framework to oversee, evaluate, and provide guidance on the implementation of necessary sanctions has been highlighted as a significant challenge [58].
(C2) Limited financial and human resources	To ensure the security of the SSCS, it is essential to involve highly qualified and well-educated cybersecurity experts in the process. Since it is impossible to predict when and where attacks may occur, these experts must continuously monitor the systems 24/7 and respond to threats in real time. This is considered a significant challenge, due to both the current shortage of qualified personnel and the substantial economic resources required. Moreover, when time pressure is added as a third factor, limited resources lead institutions to postpone investments in preventive measures related to SSC security and delay the development of valid security protocols.
(C3) Weak configuration management practices	From the viewpoint of an attacker aiming to compromise a target system, the process typically starts by locating known vulnerabilities or insecure configuration patterns within the system’s codebase, including elements specified through infrastructure as code [59]. These discovered weaknesses serve as a foundation for mapping out potential attack vectors, formulating tailored exploitation strategies, and ultimately executing a successful intrusion [59].
(C4) Insecure software distribution mechanisms	If insecure protocols exist in the processes and pathways followed by components of the holistic software—from the developer to the end or intermediate user—or if the process evolves in a way that is difficult to control, this situation presents a significant challenge.
(C5) Human factors	Although industrialization and automation are advancing rapidly worldwide, the creation, development, and management of technology and software ultimately depend on human involvement. In any process that includes human participation, there is a risk of unethical behavior, intentional code or data leaks, or the insertion of malicious software components into otherwise structured code [60]. Additionally, the lack of training or insufficient awareness of personnel regarding software security can further exacerbates the risks associated with human factors.
(C6) Inadequate continuous monitoring and incident response capabilities	Focusing primarily on past attacks and known vulnerabilities—while neglecting to proactively address potential new attack vectors—can result in being unprepared for emerging threats. Additionally, the lack of robust processes for continuously monitoring the SBOM and the entire software supply chain, including its dependencies, and for responding promptly when an attack is detected, represents a significant challenge for SSC security [61].
(C7) Insufficient authorization and access control measures	One of the key enablers of ethical violations related to human factors is the failure to properly establish authorization and access control protocols, which can make systems more vulnerable to exploitation. Even a small gap or leak in the system can lead to significant and far-reaching consequences [60].
(C8) Open-source use and missing SBOM	Although the use of open-source code has positively contributed to advancements in the software sector, its openness also introduces security risks. In particular, tracing the origin of attacks involving open-source components is often difficult [31,36]. The absence of a SBOM is critical, as it hinders the ability to track all components within a software project and to identify their sources [61]. While the adoption of SBOM is becoming more widespread, secondary challenges—such as documentation issues, lack of standardized formats, and the fact that not all open-source code includes an SBOM—complicate the monitoring, management, and execution of secure software processes [23,29].
(C9) Vulnerabilities linked to dependency usage and update procedures	Secure connection and then not checking this secure connection at all can cause these points to be vulnerable to attacks. The selection and visibility of dependencies can also be considered a challenge because it causes a kind of security breach. When updates are made and what checks they are passed through are also critical because in the studies conducted, it has been observed that especially during these updates, malicious software has the opportunity to enter the system [24].
(C10) Growing complexity and diversity of cyber-attacks further exacerbate the threat landscape.	The uncertainty surrounding which part of the entire process will be attacked, when, and how—combined with the increasing sophistication of attack techniques—makes it difficult to maintain trust in the process. Additionally, weak SBOM traceability further exacerbates this challenge [33].

The study does not merely repeat standard SBOM-related issues. While SBOM challenges were certainly considered, our work also incorporates a broader set of software supply chain security challenges identified through a detailed review of the literature and expert input. This includes both SBOM-specific and non-SBOM-specific factors, such as Lack of standardized regulations and industry-wide security protocols, Limited financial and human resources, Weak configuration management practices, Insecure software distribution mechanisms, Human factors, Insufficient authorization and access control measures. By applying the DEMATEL method, we not only categorize these challenges but also uncover the causal relationships among them, offering a novel analytical perspective that goes beyond listing known issues. In the application section, the previously explained steps of the DEMATEL method were implemented to assess the security of the SSC factors.

**Step 1.** First, the initial direct-relation matrix Z was created, as shown in Table 4, with all main diagonal elements set to zero. The direct relationship matrix used in this study was derived from a collaborative evaluation conducted by software experts with substantial industry and academic experience in a highly specialized and emerging application domain. Due to the niche nature of the field, the pool of qualified experts was inherently limited. The expert group was selected based on predefined criteria, including a minimum of 10 years of professional experience and active involvement in software development and decision-making processes related to SSC. The expert group consisted of seven members, comprising two academic experts and five industry practitioners. Among them, three were software specialists with direct expertise in SSC-related systems. Expert opinions were collected through structured workshops, during which participants collaboratively evaluated, discussed, and reached consensus on the interactions among the defined dimensions. The inclusion of both academic and industry perspectives was intended to enhance the reliability and practical relevance of the derived relationship matrix. Given the expert-based nature of the methodology and the exploratory focus of the study, the selected panel is considered sufficient to provide informed and reliable judgments. A consensus-driven approach was employed to establish agreement on the relationships within the direct relationship matrix.

**Table 4:** The group direct-relation matrix *Z.*

	C1	C2	C3	C4	C5	C6	C7	C8	C9	C10
C1	0	1	3.2	2.8	1.2	2.4	3.6	2.3	1.4	0.7
C2	0	0	2.2	3	1.7	4	1.6	1	2.2	1.8
C3	1	0	0	2	0	3.4	2.9	2.5	3.3	2.6
C4	1.3	1.7	2.3	0	1.3	3.2	3	2.8	3.1	4
C5	2	1	2.1	2.7	0	2.7	1	1	3	1.8
C6	1.5	1.2	2.5	3.6	1.4	0	3.1	2	4	4
C7	2.4	0	3	3.2	1	2.8	0	1	1	2
C8	1.7	1	2.4	4	0	4	3	0	3.4	4
C9	2.7	1	1.3	3.4	1	3	2.5	1.7	0	3
C10	2	2.7	2.6	3.4	2	3.3	2.8	2.7	3.5	0

**Step 2.** The value of *s* was determined through the calculation 1/25, resulting in *s* = 0.04. Using the Z matrix, the normalized direct-relation matrix *D* was subsequently generated, as shown in Table 5.

**Table 5:** The normalized direct-relation matrix D.

	C1	C2	C3	C4	C5	C6	C7	C8	C9	C10
C1	0	0.04	0.13	0.11	0.05	0.09	0.14	0.09	0.05	0.03
C2	0	0	0.09	0.12	0.07	0.16	0.06	0.04	0.09	0.07
C3	0.04	0	0	0.08	0	0.14	0.11	0.1	0.13	0.10
C4	0.05	0.07	0.09	0	0.05	0.13	0.12	0.11	0.12	0.16
C5	0.08	0.04	0.08	0.11	0	0.11	0.04	0.04	0.12	0.07
C6	0.06	0.05	0.1	0.14	0.05	0	0.12	0.08	0.16	0.16
C7	0.09	0	0.12	0.13	0.04	0.11	0	0.04	0.04	0.08
C8	0.07	0.04	0.09	0.16	0	0.16	0.12	0	0.13	0.16
C9	0.11	0.04	0.05	0.14	0.04	0.12	0.10	0.06	0	0.12
C10	0.08	0.11	0.10	0.14	0.08	0.13	0.112	0.11	0.14	0

**Step 3.** By utilizing the formulation of D (I − D) ^−1^, the total-relation matrix 𝑇 was calculated (see Table 6).

**Table 6:** The total-relation matrix *T.*

	C1	C2	C3	C4	C5	C6	C7	C8	C9	C10
C1	0.2676	0.2098	0.4835	0.5705	0.2140	0.5599	0.5455	0.3949	0.4794	0.4615
C2	0.2631	0.1744	0.4347	0.5665	0.2338	0.6020	0.4621	0.3416	0.5041	0.4930
C3	0.3120	0.1806	0.3656	0.5484	0.1743	0.5927	0.5239	0.4039	0.5476	0.5311
C4	0.3844	0.2862	0.5386	0.5880	0.2648	0.7025	0.6234	0.4874	0.6451	0.6782
C5	0.3305	0.2091	0.4249	0.5460	0.1655	0.5469	0.4361	0.3371	0.5183	0.4783
C6	0.3994	0.2733	0.5531	0.7245	0.2728	0.5987	0.6369	0.4692	0.6834	0.6878
C7	0.3329	0.1645	0.4453	0.5424	0.1952	0.5306	0.3839	0.3281	0.4330	0.4684
C8	0.4139	0.2733	0.5637	0.7553	0.2299	0.7550	0.6522	0.4083	0.6803	0.7072
C9	0.3902	0.2343	0.4499	0.6332	0.2286	0.6190	0.5444	0.4033	0.4640	0.5751
C10	0.4303	0.3349	0.5816	0.7501	0.3045	0.7494	0.6534	0.5110	0.6961	0.5761

**Step 4.** Following the computation of the T matrix, the r, c, (r + c), and (r − c) matrices were also calculated. The values of these matrices are presented in Table 7.

**Table 7:** r, c, (r + c) and (r-c) matrices.

	r	c	r + c	r-c	Role
**C1**	4.186	3.524	7.710	0.662	cause
**C2**	4.075	2.340	6.415	1.735	cause
**C3**	4.180	4.840	9.02	−0.660	effect
**C4**	5.198	6.224	11.423	−1.026	effect
**C5**	3.992	2.283	6.276	1.709	cause
**C6**	5.299	6.256	11.555	−0.957	effect
**C7**	3.824	5.461	9.286	−1.637	effect
**C8**	5.439	4.084	9.523	1.354	cause
**C9**	4.541	5.651	10.193	−1.109	effect
**C10**	5.587	5.656	11.244	−0.069	effect

The *(C1) Lack of standardized regulations and industry-wide security protocols*, *(C2) Limited financial and human resources*, *(C5) Human factors* and *(C8) Open-source use and missing SBOM factors*, where (R-C) has positive values, exhibit a net influence on the other factors and can be classified as the cause group.

Conversely, *(C3) Weak configuration management practices*, (*C4) Insecure software distribution mechanisms*, *(C6) Inadequate continuous monitoring and incident response capabilities*, *(C7) Insufficient authorization and access control measures*, *(C9) Vulnerabilities linked to dependency usage and update procedures* and *(C10) Growing complexity and diversity of cyber-attacks* are influenced by the other factors and should be classified as the effect group.

**Step 5.** The threshold of 0.4632, corresponding to the average of the values in the T matrix, was utilized to analyze relationship among the challenges. Values in the T matrix that exceed this threshold are highlighted in color in Table 8.

**Table 8:** T matrix colored based on the threshold: 0.4632 (*).

	C1	C2	C3	C4	C5	C6	C7	C8	C9	C10
C1	0.2676	0.2098	0.4835	0.5705	0.2140	0.5599	0.5455	0.3949	0.4794	0.4615
C2	0.2631	0.1744	0.4347	0.5665	0.2338	0.6020	0.4621	0.3416	0.5041	0.4930
C3	0.3120	0.1806	0.3656	0.5484	0.1743	0.5927	0.5239	0.4039	0.5476	0.5311
C4	0.3844	0.2862	0.5386	0.5880	0.2648	0.7025	0.6234	0.4874	0.6451	0.6782
C5	0.3305	0.2091	0.4249	0.5460	0.1655	0.5469	0.4361	0.3371	0.5183	0.4783
C6	0.3994	0.2733	0.5531	0.7245	0.2728	0.5987	0.6369	0.4692	0.6834	0.6878
C7	0.3329	0.1645	0.4453	0.5424	0.1952	0.5306	0.3839	0.3281	0.4330	0.4684
C8	0.4139	0.2733	0.5637	0.7553	0.2299	0.7550	0.6522	0.4083	0.6803	0.7072
C9	0.3902	0.2343	0.4499	0.6332	0.2286	0.6190	0.5444	0.4033	0.4640	0.5751
C10	0.4303	0.3349	0.5816	0.7501	0.3045	0.7494	0.6534	0.5110	0.6961	0.5761

(*) Criterion names highlighted in green represent the dominant criteria, while values shown in gray indicate those exceeding the threshold.

**Fig 1:**
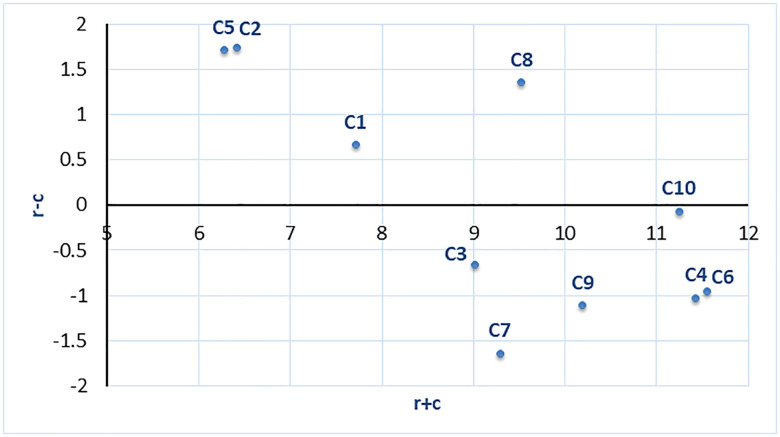
Cause and Effect Diagram.

Each security challenge is positioned according to its prominence (r + c) and net influence (r-c) values and these positions help distinguish between cause-and-effect groups (see Fig 1). In the diagram, (r + c) line shows providing insight into prominence, indicating how much a criterion affects other criteria (r) and how much it is affected by others (c). In other words, a high value suggests that the criterion plays an important role in the overall system. (r-c) line providing information about the relation. If this value is positive, the criterion belongs to the cause group, meaning it influences other criteria. Conversely, if the r − c value is negative, the criterion is part of the effect group, meaning it is influenced by other criteria. Challenges included in the Cause Group (r − c > 0) are the effective (cause) factors. *(C1) Lack of standardized regulations and industry-wide security protocols*, *(C2) Limited financial and human resources*, *(C5) Human factors* and *(C8) Open-source use and missing SBOM* fall into this group. *(C2) Limited financial and human resources, and (C5) Human factors*: these are challenges with both high r − c and medium r + c values. Thus, they can be considered effective and important challenges within the system. **(***C1) Lack of standardized regulations and industry-wide security protocols and (C8) open-source use and missing SBOM*: These elements have positive r − c values, indicating that they influence other challenges, but they are less dominant in terms of overall prominence. According to the results, the budget allocated to security expenditures can be considered a key trigger for the entire process. This triggering effect is further reinforced by human factors. Among the cause factors, (*C2) Limited financial and human resources, and (C5) Human factors* stand out as the primary drivers with strong influence on the system. *(C1) Lack of standardized regulations and industry-wide security protocols* and *(C8) Open-source use and missing SBOM* are moderately influential and are among the factors that affect other criteria to a certain extent.

On the other hand, the result factors, *(C3) Weak configuration management practices, (C4) Insecure software distribution mechanisms, (C6) Inadequate continuous monitoring and incident response capabilities, (C7) Insufficient authorization and access control measures, (C9) Vulnerabilities linked to dependency usage and update procedures, (C10) Growing complexity and diversity of cyber-attacks* are influenced by other criteria within the system. Within this group, *(C4) Insecure software distribution mechanisms, (C6) Inadequate continuous monitoring and incident response capabilities and (C10) Growing complexity and diversity of cyber-attack*s are particularly notable, as they are both highly affected and play a critical role in the system. *(C4) Insecure software distribution mechanisms* and *(C6) inadequate continuous monitoring and incident response capabilities exhibit* high r + c values but negative r − c values. This indicates that these challenges are both critical and heavily influenced within the system. Accordingly, C4 and C6 can be regarded as the primary areas where system weaknesses become most evident, suggesting that targeted and precautionary mechanisms should be strategically established to address these challenges. *(C3) Weak configuration management practices, (C7) Insufficient authorization and access control measures, (C9) Vulnerabilities linked to dependency usage and update procedures*: These challenges have low r + c and negative r − c values. This places them in the group of less important and affected factors within the system.

*(C10) Growing complexity and diversity of cyber-attacks* has high prominence (r + c) but a negative net effect (r − c). In other words, it is a highly important challenge that is significantly influenced by external factors. When the interaction between challenges is examined overall, criteria with positive r − c values, such as *(C2) Limited financial and human resources and (C5) Human factors* can be considered focal points for improving the security of the system, as they influence other criteria. Therefore, it would be logical to address these challenges as a first priority. Criteria with negative r − c values and high r + c values, such as *(C4) Insecure software distribution mechanisms, (C6) Inadequate continuous monitoring and incident response capabilities* and *(C10) Growing complexity and diversity of cyber-attacks* can be addressed as a secondary priority. Given their critical role in the system’s security, it would be appropriate to implement additional measures to monitor and manage these challenges effectively.

Considering all these aspects, initiatives aimed at improving C5 (human factors) and C2 (financial and human resource constraints) are likely to generate a cascading effect, leading to broader and more sustainable improvements in the overall security performance of the system. Because these challenges function as primary drivers within the causal structure, interventions targeting them can indirectly mitigate multiple dependent vulnerabilities across the software supply chain. From a legislative and decision-making perspective, policies and strategies that prioritize investments in human capital, training, awareness, and adequate security budgeting are expected to produce cumulative benefits and play a pivotal role in strengthening SSC security in a holistic and long-term manner. Furthermore, C8, frequently highlighted in the literature as a critical challenge, is positioned within the cause group, representing issues related to open-source usage and missing SBOMs. From this perspective, C8, similar to human factors and financial constraints, functions as a triggering factor within the system. The widespread use of open-source code in software projects, while often unavoidable, introduces inherent vulnerabilities that increase exposure to security threats. Accordingly, this challenge exhibits behavioral characteristics comparable to those of human-related factors and can be considered one of the system’s primary triggers. Since policymakers cannot realistically manage SSC security by restricting the use of open-source software, attention should instead be directed toward other interrelated factors within the system. In particular, strengthening human resources, enhancing governance structures, and implementing robust control and monitoring mechanisms can help mitigate the risks associated with open-source dependencies and improve the overall security performance of the system.

**Fig 2:**
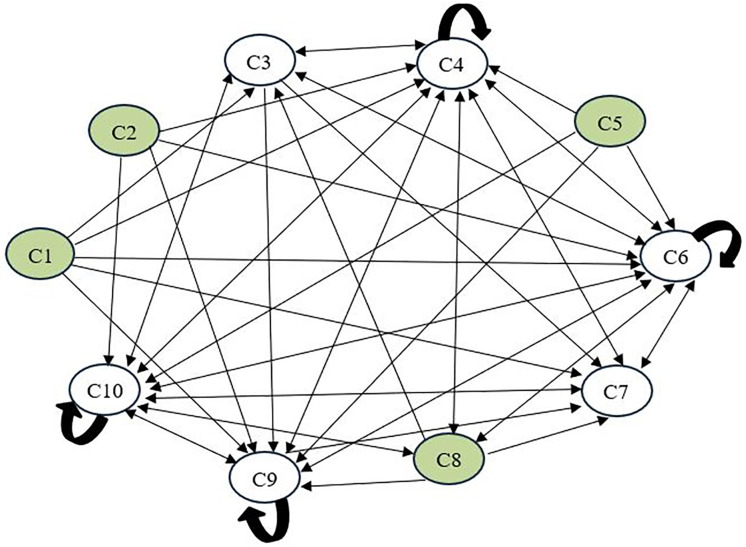
IRM based on the threshold: 0.4632.

The interrelationships and direction of influence among the challenges are visually presented in Fig 2. Arrows at the graphics shows the effect of one criterion on another. For example, if there is an arrow going from C2 to C6, it means that C2 has an effect on C6. If there are arrows in both directions, it indicates a two-way interaction.

According to Fig 2, (*C1) Lack of standardized regulations and industry-wide security protocols, (C2) Limited financial and human resources* and *(C5) Human factors* are challenges that only send arrows and do not receive any arrows. This shows that these challenges are critical challenges that affect the system in general. *(C4) Insecure software distribution mechanisms, (C6) Inadequate continuous monitoring and incident response capabilities, (C7) Insufficient authorization and access control measures, (C9) Vulnerabilities linked to dependency usage and update procedures* and *(C10) Growing complexity and diversity of cyber-attacks* are challenges in the group that are generally affected by other challenges. (*C4) Insecure software distribution mechanisms, (C6) Inadequate continuous monitoring and incident response capabilities, (C9) Vulnerabilities linked to dependency usage and update procedures* and *(C10) Growing complexity and diversity of cyber-attacks* are also challenges that have internal dependencies. This indicates that these challenges should not be considered in isolation, but rather evaluated collectively along with the other challenges that influence them.

*(C3) Weak configuration management practices* has mutual interactions with challenges *(C4) Insecure software distribution mechanisms, (C6) Inadequate continuous monitoring and incident response capabilities* and *(C10) Growing complexity and diversity of cyber-attacks.*

*(C4) Insecure software distribution mechanisms* has mutual interactions with *(C3) Weak configuration management practices*, *(C6) Inadequate continuous monitoring and incident response capabilities*, *(C7) Insufficient authorization and access control measures*, (*C8) Open-source use and missing SBOM, (C9) Vulnerabilities linked to dependency usage and update procedures* and *(C10) Growing complexity and diversity of cyber-attacks.*

*(C6) Inadequate continuous monitoring and incident response capabilities* has mutual interactions with challenges *(C3) Weak configuration management practices, (C4) Insecure software distribution mechanisms, (C7) Insufficient authorization and access control measures*, (*C8) Open-source use and missing SBOM, (C9) Vulnerabilities linked to dependency usage and update procedures* and *(C10) Growing complexity and diversity of cyber-attacks.*

*(C7) Insufficient authorization and access control measures* has mutual relationship with challenges *(C4) Insecure software distribution mechanisms, (C6) Inadequate continuous monitoring and incident response capabilities, (C10) Growing complexity and diversity of cyber-attacks.*

(*C8) Open-source use and missing SBOM* has mutual relationships with *(C4) Insecure software distribution mechanisms*, *(C6) Inadequate continuous monitoring and incident response capabilities, (C10) Growing complexity and diversity of cyber-attacks.*

*(C9) Vulnerabilities linked to dependency usage and update procedures* has mutual interactions with challenges *(C4) Insecure software distribution mechanisms, (C6) Inadequate continuous monitoring and incident response capabilities, (C10) Growing complexity and diversity of cyber-attacks.*

*(C10) Growing complexity and diversity of cyber-attacks* has mutual interactions with *(C3) Weak configuration management practices*, *(C4) insecure software distribution mechanisms, (C6) Inadequate continuous monitoring and incident response capabilities, (C7) Insufficient authorization and access control measures*, (*C8) Open-source use and missing SBOM, (C9) Vulnerabilities linked to dependency usage and update procedures.*

**Fig 3:**
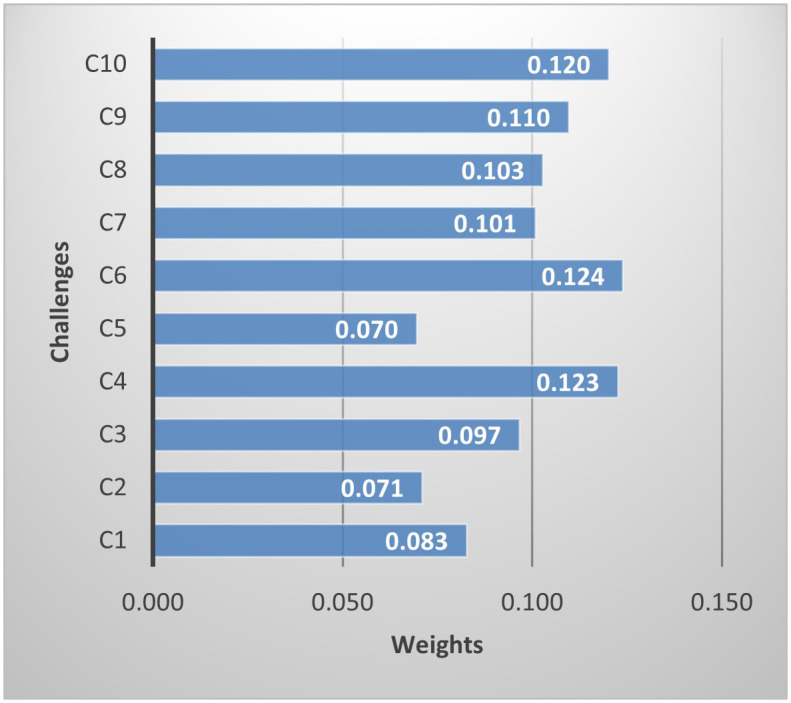
The importance degree of challenges.

The calculated weights of the challenges are presented in Fig 3. *(C4) Insecure software distribution mechanism*, *(C6) Inadequate continuous monitoring and incident response capabilities* and *(C10) Growing complexity and diversity of cyber-attacks* are the three most important challenges.

Case analyses have been conducted on the well-known and globally impactful SolarWinds case, highlighting that one of the primary causes was the introduction of malware and data leakage through software updates [62]. Specifically, C9 challenge, which examines the impact of updates on large-scale software projects, was found to exhibit internal dependencies in the obtained results. Its ranking as the fourth most critical challenge in the order of importance demonstrates that the study’s results are consistent with real-world cases. The issue we discussed regarding missing SBOMs and the use of open-source code, which we examined through the C8 challenge, is precisely what is observed in the Log4Shell case. Specifically, the use of open-source code combined with the lack of SBOM maintenance makes it difficult to identify traceable code components, thereby increasing vulnerability to attacks [63,64].

The Target data breach that occurred in 2013 is a well-documented example in which the company’s disregard of warnings about cyberattacks attempting to access its systems, namely insufficient monitoring and ineffective incident response (C6), allowed attackers to operate for an extended period despite numerous security alerts, thereby significantly amplifying the impact of the breach [65]. The challenge identified as C6, which directly addresses this situation, emerged as the most critical challenge based on the obtained results, drawing particular attention and being classified as a significant “effect” challenge. Several cybersecurity incidents demonstrate that malicious code can be intentionally introduced by insiders with legitimate Access [60,66], highlighting the critical role of human factors (C5) in cybersecurity risk management. Logic bomb cases [66], in particular, illustrate how employees can deliberately embed harmful functionality into software systems, underscoring the importance of effective human resources practices such as background checks, access control aligned with job roles, and employee offboarding processes in mitigating insider threats.

## Discussion

The goal of this research was threefold: to identify the primary challenges impacting SSC security, to analyze the relationships between these challenges, and to establish which ones act as root causes versus resulting effects. To answer the first research question (RQ1), the study used a combination of an extensive literature review and expert input to pinpoint ten major challenges. These challenges fall across regulatory, organizational, operational, and technical areas, highlighting the complex nature of SSC security where vulnerabilities stem from a mix of human and technological factors.

At first glance, the most critical insight from the DEMATEL analysis is the classification of challenges into distinct groups and the rationale behind this grouping. In response to RQ2, the DEMATEL results provided a clear separation between those challenges that act as drivers (causes) and those that are outcomes (effects) in the SSC system. This classification is particularly important as it highlights the central challenges within the system. Specifically, Lack of standardized regulations, limited financial and human resources, Human factors, Open-source use, and missing SBOM are categorized within the cause group. These challenges exert considerable influence on other factors while comparatively less affecting themselves. C2 (Limited financial and human resources) and C5 (Human factors) stand out due to their high impact potential and moderate overall prominence within the system. This positioning designates them as primary targets for strategic intervention. In the security context in SSC, addressing these two foundational issues—through resource allocation and human-centered strategies—could produce a cascading positive effect on the broader set of challenges. The findings for RQ3 emphasize that C2 (Limited financial and human resources) and C5 (Human factors) are the dominant causal elements driving SSC insecurity. Their significant net influence and prominence values mean that interventions focused on improving human capital and organizational resources will serve as the most effective leverage points for achieving broad security improvements.

Surprisingly, the highly critical challenges—C4 (Insecure software distribution mechanisms), C6 ((Inadequate continuous monitoring and incident response capabilities), and C10 (Growing complexity and diversity of cyber-attacks)—might have been expected to serve as the main drivers, i.e., the root causes of security challenges within the software supply chain system, at the outset of the study. However, the findings revealed that these challenges were classified within the effect group, which is somewhat counterintuitive. While high-impact issues like C4, C6, and C10 could reasonably be assumed to act as internal triggers within the system, the results obtained through DEMATEL suggest that they are actually symptoms of deeper organizational and structural problems, particularly resource constraints and human factors, rather than primary causes. The findings obtained in this study become more meaningful when considered together with established frameworks for Software Supply Chain (SSC) security. For instance, NIST SP 800−161 Rev.1 identifies governance, resource allocation, human factors, and supplier oversight as fundamental pillars of supply chain risk management [67]. From this perspective, the emergence of challenges C2 (Limited financial and human resources) and C5 (Human factors) as dominant causal elements in this study directly aligns with NIST’s view that security weaknesses may often stem from organizational and managerial deficiencies rather than purely technical flaws. A noteworthy aspect here is that NIST is not a mandatory framework that all organizations worldwide are required to adopt.

From another angle, challenges C4 (Insecure software distribution mechanisms), C6 (Insufficient continuous monitoring and incident response capabilities), and C7 (Insufficient authorization and access control measures) appear in the impact group of the results, reflecting behavior that is broadly consistent with the principles of Zero Trust Architecture (ZTA). However, the requirement for mandatory controls on every request may introduce time and performance overhead in ZTA-based systems [68]. In this regard, a trade-off emerges between systems that are highly secure but cumbersome, and those that are acceptably secure, faster, yet occasionally exposed to threats—an issue that manifests itself as the challenges discussed in this paper. The Zero Trust approach treats monitoring, verification, and secure distribution mechanisms not as independent controls, but as natural outcomes of identity management, process maturity, and corporate governance [69,70]. Within this context, weaknesses in monitoring and incident response can be interpreted as consequences of the lack of skilled human resources and insufficient sustainable investment, both of which are emphasized in this study.

Furthermore, the absence of a Software Bill of Materials (SBOM) (C9) is identified as a causal factor, and this finding is directly related to the SBOM best practices recommended by NIST [67]. The effective implementation of SBOM requires not only technical tools but also interdisciplinary coordination, skilled personnel, and adequate budget allocation. This observation supports one of the study’s core findings: that human- and resource-based challenges are prerequisites for the success of technical security controls.

In conclusion, rather than contradicting existing SSC security frameworks, the findings of this study empirically validate the underlying causal structures of these frameworks. The prominence of human factors and resource constraints as root causes demonstrates that technical controls in software supply chain security can only be effective when supported by strong governance, competent human resources, and sustained investment.

On the other hand, the IRM reveals a dense web of mutual interdependencies among challenges. This is expected in complex domains such as SSC security. However, identifying these reciprocal relationships is essential for understanding the system’s dynamics and designing effective interventions. The intensity of these interactions suggests that tackling challenges in isolation may not yield long-term improvements. Instead, a systemic and integrated approach is required—one that moves beyond individual solutions toward holistic security strategies capable of addressing the interrelated nature of cyber risks in the SSC environment.

As an advantage of the DEMATEL technique, it is possible to modify the threshold value in IRM to enable a more general or more detailed view of the topic, thereby generating different cause-effect diagrams based on the new thresholds. However, using the average of the values in the matrix as the threshold allows for a value to be determined without any loss of expert input, making it the most suitable option for the structure of this study. Nevertheless, if significant advancements are made in SSC security in the future, it may become appropriate to increase the threshold value and adopt a broader perspective on the challenges. At the current stage, however, observing all relationships between the challenges and identifying the cause-and-effect groups is essential.

## Conclusion

This study aimed to explore the major challenges that threaten the security of the SSC and to understand how these challenges influence one another. In recent years, software systems have become more interconnected and increasingly depend on third-party services, open-source components, and automated distribution tools. While this has improved software development speed and flexibility, it has also introduced serious risks to security. Attacks targeting the SSC are becoming more common and complex, making it crucial to identify the root causes of these vulnerabilities and the relationships between them. The DEMATEL method was applied to analyze these challenges in a structured way. This method helped to separate the challenges into two main groups: cause factors, which influence other challenges, and effect factors, which are mainly shaped by those causes. The challenges do not exist in isolation; they are part of a tightly connected system where one weakness can lead to many others. This interdependency shows that fixing one problem, such as improving monitoring, without addressing its causes, like poor staffing or lack of policies, may not lead to lasting improvements. Therefore, policymakers and organizational leaders should prioritize tackling foundational problems first. Investments in cybersecurity training, standard-setting, and proper documentation (such as SBOMs) will have a wide-reaching effect across the entire software development and distribution process.

The study uses a systems-based approach to understand how different security barriers are connected. From this perspective, a three-level framework for implementing security measures can be proposed. The first level focuses on establishing strong governance. At this stage, organizations are encouraged to develop clear security policies, assign specific resources for security tasks, and include security responsibilities in their strategic plans. These actions help ensure that security efforts are not random or short-term, but rather part of a long-term strategy that supports the organization’s overall goals. The second level involves strengthening day-to-day operational processes. This includes putting in place formal procedures for secure system configuration, managing software components consistently, and enforcing strict access controls. When these practices are integrated into everyday workflows, organizations can reduce the chances of security breaches caused by neglected or weak operational procedures. The third and final level focuses on building the ability to adapt to new and evolving threats. This requires continuous monitoring of systems, using threat intelligence, and developing flexible plans for responding to incidents. It is important to note that these three layers (governance, operations, and adaptability) should not be treated separately. When aligned, they help create a strong, proactive, and flexible security system that is better equipped to prevent the spread of risks linked to the complex web of challenges identified in the study.

Despite its strengths, this study has some limitations that should be considered. First, the method used (DEMATEL) shows the relationships between problems at one point in time, but it does not show how these problems might change over time.

At this stage, lawmakers or decision-makers cannot apply these findings in practice because the article’s results have not yet been published, making it difficult to fully assess their practical applicability and interpret the causal relationships. Most focus is on internal problems, like human error or lack of resources, while outside threats, such as hackers or advanced attacks, are not discussed in detail. In addition, the study uses challenges found in past research so that it might miss new or growing risks. Lastly, although the study shows which problems are most important, it does not clearly explain what steps organizations should take to fix them.

To improve upon these limitations, future research should consider several directions. First, future studies can involve a larger and more diverse panel of experts, including practitioners from different industries such as healthcare, finance, and government. This would improve the reliability and generalizability of the findings. Second, combining DEMATEL with other decision-making methods, such as the Analytic Network Process (ANP) or fuzzy logic, could help account for uncertainty and complexity more effectively. Third, longitudinal studies could track how these challenges evolve and how different interventions influence the system. Fourth, applying the model to real-world organizations through case studies or pilot projects would provide practical validation and help tailor the framework to specific needs. Additionally, researchers can explore the development of automated tools or dashboards based on the DEMATEL model, which organizations could use to assess and monitor SSC security in real time.

Understanding the causal structure of SSC vulnerabilities is critical for developing effective and resource-efficient security strategies, as clarifying how security challenges spread throughout the software ecosystem establishes a basis for creating evidence-based policies, enhancing organizational planning, and ultimately boosting cybersecurity resilience. Since policymakers cannot realistically manage SSC security by restricting the use of open-source software, attention should instead be directed toward other interrelated factors within the system. In this context, strengthening human resources, enhancing governance structures, and implementing robust control and monitoring mechanisms can help mitigate the risks associated with open-source dependencies and improve the overall security performance of the system. Therefore, policymakers and organizational leaders should prioritize tackling foundational problems first, as investments in cybersecurity training, standard-setting, and proper documentation such as SBOMs can have wide-reaching effects across the entire software development and distribution process.

The findings of this study further suggest the need for nationally and internationally recognized standards that define differentiated implementation and control requirements for small and large organizations within software supply chains. Such standards could incorporate scalable security controls, allowing smaller firms to adopt essential baseline measures while enabling larger organizations to implement more advanced governance and monitoring mechanisms. In particular, standardized and well-documented systems are needed to address insider-related risks, including intentional data leakage and the deliberate insertion of malicious code. Deterrent requirements such as clearly defined accountability mechanisms, role-based access controls, traceability of code contributions, and mandatory security audits could be integrated into quality assurance and compliance processes. By embedding these controls into formalized quality and certification frameworks, policymakers and standard-setting bodies can promote consistent security practices across the software ecosystem, reduce systemic vulnerabilities, and strengthen trust among supply chain stakeholders.

In summary, this study provides a clear and structured understanding of SSC security’s obstacles and how these challenges are interconnected. It emphasizes the importance of addressing foundational issues such as human and financial resources, regulation, and open-source transparency to reduce broader systemic risks. Although more work is needed to extend these findings, the study offers a valuable starting point for decision-makers, researchers, and industry leaders seeking to build more secure and resilient software ecosystems.

## Supporting information

S1 File Supplementary material _dematel –sscm. (XLSX)
